# Optimal Energy Management Framework for Truck-Mounted Mobile Charging Stations Considering Power Distribution System Operating Conditions

**DOI:** 10.3390/s21082798

**Published:** 2021-04-15

**Authors:** Soi Jeon, Dae-Hyun Choi

**Affiliations:** School of Electrical and Electronics Engineering, Chung-Ang University, 84 Heukseok-ro, Dongjak-gu, Seoul 156-756, Korea; thdl1209@cau.ac.kr

**Keywords:** electric vehicle, mobile charging station, transportation network, power distribution network, mixed-integer linear programming

## Abstract

A high charging demand from many electric vehicles (EVs) at a fixed charging station (FCS) with a limited number of charging poles can increase the waiting time of EVs and yield an abnormal power grid condition. To resolve these challenges, this paper presents an optimization framework in which a mobile charging station (MCS) is dispatched to the overloaded FCS to reduce the number of waiting EVs while maintaining normal power grid operation. Compared to existing MCS scheduling methods that do not consider actual power distribution system operations, the proposed framework takes into account the (i) active/reactive power flow and consumption of EVs, (ii) reactive power capability of MCS, and (iii) voltage quality in power distribution systems. In coupled transportation and power distribution systems, the proposed algorithm conducts optimal operation scheduling of MCS for both road routing and charging and discharging, thereby leading to the reduction of waiting EVs within the allowable voltage range. The proposed MCS optimization algorithm was tested in IEEE 13-bus and 33-bus distribution systems coupled with 9-node and 15-node transportation systems, respectively. The test results demonstrate the effectiveness of the proposed algorithm in terms of number of waiting EVs, voltage magnitude deviation, and reactive power of the MCS.

## 1. Introduction

Given that traditional gasoline-powered cars generate significant greenhouse gas emissions and carbon pollution, electric vehicles (EVs) are becoming crucial entities and viable options to reduce environmental pollution progressively. Compared to gasoline vehicles, EVs result in a decrease in noise pollution and maintenance costs, as well as a reduction in greenhouse gas emissions and carbon pollution from the transportation sector [1]. In addition, efficient management for charging and discharging of EVs can enhance power grid flexibility, thereby leading to an economical power distribution grid operation. Evidently, transportation electrification (i.e., moving from gasoline vehicles to a full EV) holds promise for lowering environmental pollution and ensuring smarter operations of distribution grids [2].

However, owing to insufficient charging infrastructure, long charging times, limited driving range for EVs, and high EV battery costs, the deployment of EVs and their associated services in the transportation and power distribution networks is becoming slower. In particular, among the aforementioned limitations, the lack of fixed charging stations (FCSs) is one of the most significant challenges in moving to transportation electrification. An FCS is a fixed facility with a limited number of charging poles (CPs). FCSs are generally located near public roads, commercial buildings, and homes for EV charging and use the power received from the power distribution grid. The lack of mobility and limited discharging capability of FCSs can yield a significant increase in the waiting time for EVs at an FCS and dissatisfaction for EV drivers, as well as resulting in grid overload and instability when high charging demand with a large number of EVs occurs at FCSs. These challenges suggest the need for a more efficient charging method for EVs to ensure a satisfactory charging service and stable grid operation.

Recently, mobile charging stations (MCSs) received attention as a new technology for EV charging because they can offer charging services at any time and location. The mobility characteristics of MCSs resolve the limitations of FCSs by (i) reducing the waiting time of EVs and the EV driver’s range anxiety through the provision of additional CPs to FCSs and (ii) mitigating grid insecurity (e.g., significant voltage drop at the distribution node due to EV charging) through a charging and discharging operation of the MCS for a stable and economical grid operation from the perspective of the distribution grid operator. Furthermore, compared to FCSs, MCSs can be rapidly relocated and built up at the best locations using their mobility, thereby saving the investment cost of FCSs. MCSs are broadly categorized into (i) portable units for charging EVs from commercial/residential buildings and FCSs, (ii) utility-owned truck-mounted charging stations with large-scale energy storage systems (ESSs), and (iii) vehicle-to-vehicle charging through wireless charging without direct connection between EVs and chargers [3]. This study aimed to develop an energy management framework for truck-mounted MCSs (the aforementioned second category of MCSs) to be dispatched to overloaded FCSs and to be controlled through charging and discharging to reduce the number of waiting EVs while maintaining stable distribution grid operating conditions.

**Figure 1:**
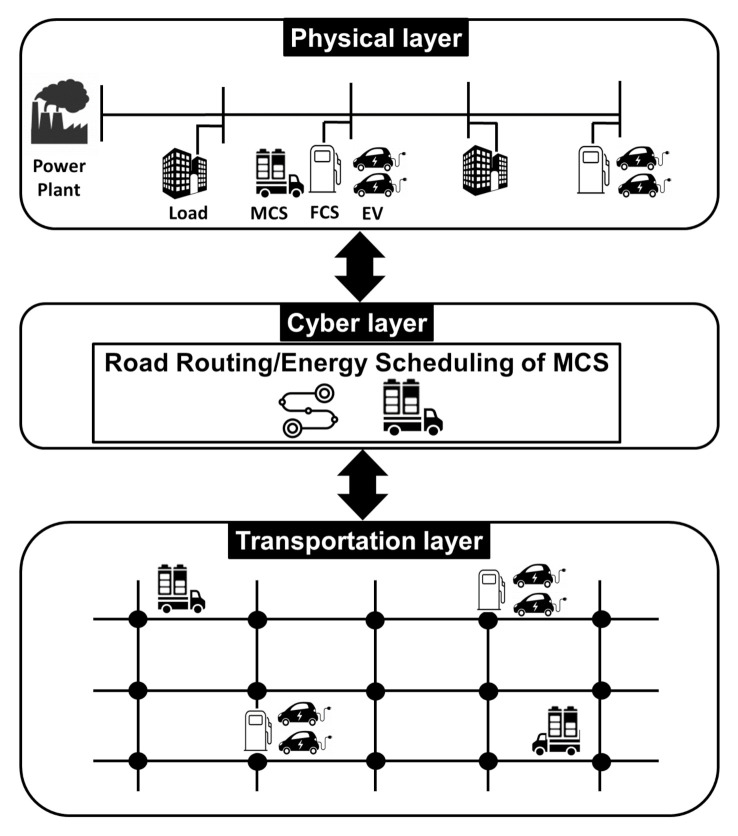
A three-layer architecture for the proposed mobile charging station (MCS) control framework.

The primary goal of this study is to present a more practical MCS optimization algorithm that can be implemented in realistic operation conditions of power distribution systems. As shown in Figure 1, the proposed algorithm is executed in a cyber-physical transportation system (CPTS) with three layers, corresponding to (i) a computing, communication, and control entity in the cyber layer; (ii) a power distribution system in the physical layer; and (iii) a road network in the transportation layer. Given a tighter coupling between the transportation system and the power distribution system [4], the primary goal of our study was to develop an optimization framework in the cyber layer that calculates the schedule of the road routing in the transportation layer and the schedule of energy charging/discharging of the MCSs in the physical layer. In the CPTS, the proposed algorithm allows the MCSs to be quickly dispatched to the desired FCSs to reduce the number of waiting EVs and maintain the normal voltage level along the power distribution feeder in real operation conditions of the distribution system. Specifically, the main contributions of this study are two-fold:

- We propose a CPTS-based MCS control framework in which the road routing and the charging and discharging of the MCSs are simultaneously scheduled to supply power to waiting EVs at FCSs while maintaining the normal operation conditions of the power distribution system. Unlike existing MCS dispatch methods that consider only active power charging and discharging of the MCS, the proposed framework minimizes the number of waiting EVs at FCSs and maintains the voltage quality through the minimization of nodal voltage deviation while considering all operational constraints of the power distribution system including active/reactive power flow and consumption, and voltage magnitude.
- We formulate the proposed MCS control algorithm as a mixed-integer linear programming (MILP) optimization problem. We present the equality and inequality constraints for the operation characteristics of the MCS involving: (i) the EV queue dynamics at FCSs with charging and waiting EVs and road routing in the transportation network, and (ii) charging and discharging behavior in the power distribution network. In addition, operational constraints of the power distribution system, such as linearized active/reactive power flow and allowable voltage magnitude range, are incorporated as the constraints of the proposed MCS optimization problem. The simulation results demonstrate that the proposed algorithm can be successfully implemented in a realistic power distribution system.

The remainder of this paper is organized as follows. Section 2 provides a literature review for our proposed approach. Section 3 introduces the system model for the proposed MCS control framework. Section 4 presents the optimization formulation of the proposed MCS control algorithm using the MILP method. The simulation results for the proposed MCS control algorithm are provided in Section 5, and the conclusions are presented in Section 6.

## 2. Related Research

In general, a truck-mounted ESS (TESS) owned by a utility is employed for two purposes, corresponding to the support of the amount of unserved load due to a blackout and the provision of charging service to EVs. A literature review related to the utilization of TESSs can be summarized in the following two categories.

- Distribution system service restoration: A truck-mounted generator (TG) or TESS, namely, a mobile emergency resource, is dispatched to facilitate efficient service restoration of a distribution power system against large area blackouts. The mobile emergency resource can be used to support power quickly to the power distribution system isolated from the main grid owing to some power system fault. In [5], a temporal-spatial TESS model including both the transportation and distribution power networks was incorporated into a post-disaster restoration problem along with network reconfigurations, and the financial benefit from the utilization of the TESS was quantified. A co-optimization framework for distribution system service restoration was presented in [6], in which the dispatch of both the repair crew and TG/TESS was jointly considered during the distribution system operation. A critical load restoration strategy was developed in [7] to determine the optimal location of mobile emergency resources along with an efficient microgrid formation and load switching sequence in distribution systems. In [8], a two-stage stochastic optimization method for dispatch of TGs was presented. The location of TGs was determined prior to a natural disaster in the first stage. The real-time allocation of TGs was conducted to restore critical loads after natural disaster strikes during the second stage. A day-ahead optimization method for energy management of a TESS was proposed in [9]. The objective was to maximize the profit of the distribution system operator while maintaining a normal voltage profile along the distribution feeder. In [10], a hybrid energy generation portfolio for mobile emergency resources equipped with photovoltaic panels, wind turbines, and ESSs was studied to reduce the implementation cost and enhance the mobility of the mobile emergency resources.
- Mobile charging for EVs: Using the mobility of TESSs, they can be used as MCSs equipped with a utility-scale ESS [11] to offer charging services at any time to EVs at FCSs located in commercial/residential buildings and public roads. Since the pioneering study in [12] reported the development of an MCS dispatching algorithm for reducing the waiting time of EVs at FCSs, a number of researchers have investigated the MCS dispatch problem in a joint model of transportation and power distribution systems. In [13], a communication protocol among charging station servers, FCSs, MCSs, and EVs for MCS dispatch was proposed to decrease the waiting time of EVs. In a scenario of EV waiting time reduction, two types of MCSs were considered: MCSs with and without ESS for charging EVs inside and outside the FCS, respectively. In a joint model of transportation and power distribution systems, a two-tier TESS scheduling method was developed in [14,15] to minimize the scheduling cost of TESSs. In this method, a TESS delivers surplus energy from a charging station with sufficient power supply to a charging station with limited capacity. The MCS operation framework developed in [14,15] was extended to a price-incentive scheme [16] to mitigate the overload of charging stations with limited capacity. In this case, the interaction between the distribution system operator and mobile energy storage was formulated as a Stackelberg game. In [17], two heuristic algorithms for the allocation of an MCS to a FCS based on the bin packing vertex coloring problem were presented to maximize the number of EVs charged at an FCS. The first algorithm charged a larger number of EVs with a low EV waiting time at the expense of higher computational complexity. The second algorithm illustrated the trade-off relationship between the number of charging EVs and the computation complexity. A MILP-based optimization problem was formulated in [18] to minimize the number of temporary charging service centers and MCSs and the operating costs and charging capacity of the MCSs. In [19], an MCS dispatch algorithm considering both the optimal location and routing of the MCS simultaneously was formulated as an MILP optimization problem to prevent the MCS dispatch algorithm from yielding a suboptimal solution resulting from calculating the optimal location and routing of the MCS separately. A Lyapunov-based online distributed algorithm was presented in [20]. In this algorithm, the energy management of MCSs with different power sources, such as traditional and renewable power, is conducted in an Internet-of-Things environment to maximize the long-term average profits of MCSs. In [21], a new MCS model that has a hybrid storage resource (e.g., battery, fuel cell, and ultracapacitor) was proposed to charge EVs that stay in urban and resort areas. The operation characteristics of the proposed MCS model were investigated using Simulink model. More recently, a novel charging reservation scheme and communication protocol for MCS dispatch was presented in [22] along with an accurate estimation of EV charging demands under practical constraints, such as limited chargers and spatial-temporal variations of chargers and charging demands. In [23], several heuristic approaches based on the MILP optimization problem were proposed for the MCS to charge a number of EVs at as many parking lots as possible and return to the depot where the MCS parks and charges its battery after finishing the charging service.

Our study belongs to the second category of the aforementioned literature review. However, a considerable amount of recent studies on MCS dispatching methods were conducted based on unrealistic distribution system operation conditions. In particular, the following aspects were not taken into account: (i) active and reactive power flows along with reactive power consumption in load, (ii) nodal voltage quality in terms of voltage magnitude limit, and (iii) reactive power capability of MCS. Furthermore, previous studies did not present a dynamic queue model for charging and waiting EVs at FCSs under the operation of MCSs nor did they evaluate the impact of MCSs on the efficiency of power distribution system operation.

## 3. System Model

We consider a situation in which multiple MCSs are dispatched to multiple FCSs through a transportation network for charging EVs that wait at FCSs in a power distribution network. We assume that each MCS is a truck-mounted EV equipped with a utility-scale ESS. There are two types of nodes: (i) the set of nodes I in the transportation network and (ii) the set of buses B in the power distribution network. In this study, nodes and buses are denoted by the beginning and ending points in the transportation and power distribution networks, respectively. Depending on whether the FCSs are connected to the nodes and buses in the transportation and power distribution networks, the sets I and B are decomposed into the following two subsets: $I=I^{\mathrm{FCS}}\cup I^{\mathrm{Non-FCS}}$ and $B=B^{\mathrm{FCS}}\cup B^{\mathrm{Non-FCS}}$. Given that the FCSs are located at the intersections of the transportation and power distribution networks, the set $I^{\mathrm{FCS}}$ is equal to the set $B^{\mathrm{FCS}}$. S and T represent the set of MCSs and MCS scheduling periods, respectively. The number of elements in a set A is denoted by |A|.

We assume that a distribution system operator (DSO) owns a depot where MCSs with identical battery capacities are parked and charged prior to their dispatch. The DSO has a central server that performs an optimal day-ahead operation scheduling of each MCS s∈S to reduce the number of waiting EVs while maintaining a normal distribution system operation. We also assume that the central server has all input data prior to the optimal MCS operation scheduling. These input data are categorized into fixed and predicted parameters. The former includes the number of charging poles in FCS $i\in I^{\mathrm{FCS}}$ ($N_{i}^{\mathrm{FCS},\mathrm{pole}}$), the number of charging poles in an MCS *s* ($N_{s}^{\mathrm{MCS},\mathrm{pole}}$), the charging rates of the FCS ($R_{b}^{\mathrm{FCS}}$) and MCS ($R_{b}^{\mathrm{MCS}}$) at bus $b\in B^{\mathrm{FCS}}$, the topology of the transportation and power distribution networks, and parameters for the power distribution network (e.g., resistance ($r_{hb}$) and reactance ($x_{hb}$) of the distribution line between buses *h* and *b*). The latter includes the predicted traveling time (${\hat{\gamma }}_{ij}$) of the MCS between nodes *i* and *j*, the predicted number of EVs (${\hat{N}}_{i,t}^{\mathrm{Non-MCS}}$) that arrive at FCS *i* prior to the dispatch of the MCSs, and the predicted active and reactive power consumption (${\hat{P}}_{b,t}^{\mathrm{non-EV}\;\mathrm{load}},\;{\hat{Q}}_{b,t}^{\mathrm{non-EV}\;\mathrm{load}}$) for the non-EV load at bus *b* and time *t*. We consider a situation in which the power distribution and transportation systems have smart meters and traffic sensors to monitor active/reactive power consumption and road traffic, respectively. A smart meter is an advanced smart energy sensor that monitors the real-time energy usage of the consumer through the advanced metering infrastructure and provides the energy usage data to the consumer and DSO for achieving energy saving and efficient power distribution system operation. Smart traffic sensors are used to collect road traffic information, which can be supplied to the DSO for scheduling the routing of MCSs. A prediction algorithm is assumed to accurately predict those power consumptions and road traffic condition using the sensor data. The predicted values are fed into the proposed optimization algorithm every day prior to the dispatch of MCSs.

**Figure 2:**
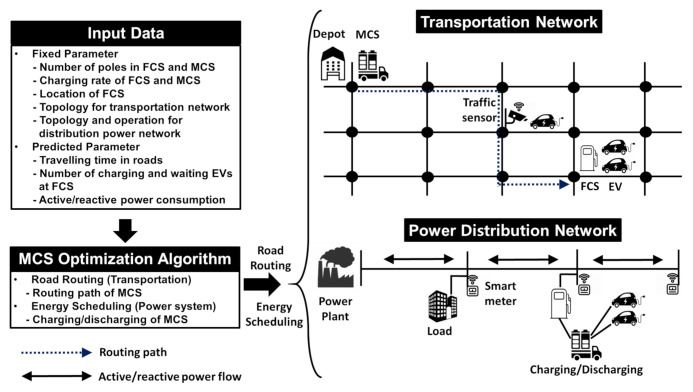
Conceptual system model for the proposed MCS control framework.

As shown in Figure 2, the proposed MCS optimization algorithm executes the following two tasks simultaneously: (1) road routing scheduling of the MCS in the transportation network and (2) active and reactive power charging/discharging scheduling of the MCS in the power distribution network. Given the aforementioned input data, the first task of the proposed approach is to calculate an optimal path schedule through which the MCSs are dispatched to the desired FCSs to minimize the number of waiting EVs at the FCS. The second task of the proposed algorithm is to conduct an optimal scheduling of the MCS charging from the power grid and MCS discharging to the waiting EVs at the FCS and the power grid while ensuring a normal voltage profile.

## 4. Proposed MCS Control Algorithm

A method that conducts the optimal routing and charging/discharging scheduling of MCSs under distribution system operating conditions is formulated as an MILP optimization problem with the following multi-objective function and constraints.

### 4.1. Objective Function

For each node i∈I and bus b∈B with scheduling period t∈T:={1,…,T}, the multi-objective function (1) for the MCS scheduling problem consists of two terms, each of which with different decision variables ($N_{i,t}^{w},V_{b,t}$):

(1) $$\min_{N_{i,t}^{w},V_{b,t}}\underset{J_{1}\left(N_{i,t}^{w}\right)}{\underbrace{\omega_{1}\sum_{t\in T}\sum_{i\in I^{\mathrm{FCS}}}N_{i,t}^{w}}}+\underset{J_{2}\left(V_{b,t}\right)}{\underbrace{\omega_{2}\sum_{t\in T}\sum_{b\in B}\left|V_{b,t}-V^{\mathrm{ref}}\right|}}.$$

The first term $J_{1}\left(N_{i,t}^{w}\right)$ is the total number of waiting EVs at all FCSs during the entire scheduling period *T*. The second term $J_{2}\left(V_{b,t}\right)$ is the total deviation of the voltage magnitude $V_{b,t}$ for bus *b* at time *t* from the voltage magnitude reference $V^{\mathrm{ref}}$ ($V^{\mathrm{ref}}$ = 1.0 p.u.). The second term $J_{2}$ is associated with the voltage quality in power distribution system. Here, voltage quality refers to the keeping of nodal voltage magnitude around the voltage magnitude reference (1.0 p.u.) within the allowable voltage range [0.95 p.u., 1.05 p.u.]. The minimization of $J_{2}$ guarantees reliable power distribution system operations from the perspective of voltage quality. The formulated optimization problem aims to minimize the number of waiting EVs ($J_{1}$) at FCS in the transportation system while maintaining the voltage quality ($J_{2}$) in the power distribution system. The non-negative parameters $\omega_{1}$ and $\omega_{2}$ are penalty weights for the reduction of waiting EVs and flattening of the nodal voltage magnitude, respectively, where $\omega_{1}+\omega_{2}=1$. These weights determine the relative importance of the number of waiting EVs at the FCSs relative to the voltage deviation. For instance, a larger $\omega_{1}$ further decreases the number of waiting EVs at the expense of less flattening of the voltage magnitude due to a smaller $\omega_{2}$. Note that $J_{1}$ and $J_{2}$ have different units. Thus, they need to be normalized for a fair impact analysis of the weights. To resolve this issue, $J_{1}$ and $J_{2}$ are normalized by ${\hat{J}}_{1}$ and ${\hat{J}}_{2}$, respectively. Here, ${\hat{J}}_{1}$ and ${\hat{J}}_{2}$ are calculated as ${\hat{J}}_{1}=\max\left(N_{i,t}^{w}\right)\times \left|I^{\mathrm{FCS}}\right|\times T$ and ${\hat{J}}_{2}=\max\left|V_{b,t}-V^{\mathrm{ref}}\right|\times \left|B\right|\times T$, respectively. The equality and inequality constraints for the MCS scheduling problem are categorized into the three subsequent subsections: operation characteristics of MCS, including the routing and charging/discharging of the MCS (Section 4.2); queue dynamics for charging and waiting EVs at the FCS (Section 4.3); and distribution system operating conditions including the active/reactive power flow equation and reactive power capability of the MCS (Section 4.4).

### 4.2. Operation Characteristics of MCSs

#### 4.2.1. Status of Nodal Visit, Traveling, and Charging/Discharging for MCS

The binary decision variable $b_{i,t,s}^{v}$ determines the status for the visit of MCS *s* at node *i* and scheduling time *t*. The visiting status includes the following two actions of the MCS: (i) arrival at node and (ii) stay with or without charging and discharging (i.e., idle status). The binary decision variables $b_{i,t,s}^{\mathrm{ch}}$ and $b_{i,t,s}^{\mathrm{dch}}$ determine the charging and discharging statuses of MCS *s* at node *i* and scheduling time *t*, respectively. The binary decision variable $b_{t,s}^{\mathrm{tr}}$ determines the traveling status of MCS *s* at scheduling time *t*. Equation (2) ensures that each MCS can at most visit one node at time *t*. Equation (3) states that no additional MCS can be dispatched and charged through the pole in FCS *i* at scheduling time *t* when the sum of the number of charging EVs and charging MCSs is larger than the number of poles in the FCS. It ensures that the MCS does not interfere with EV charging at the FCS. According to Equations (4) and (5), the charging and discharging of the MCS can be allowed only when the MCS does not travel on the transportation network at scheduling time *t* (i.e., $b_{t,s}^{\mathrm{tr}}=0$). Equation (6) guarantees that the charging and discharging of the MCS are mutually exclusive at scheduling time *t*, and no charging or discharging occurs (i.e., $b_{i,t,s}^{\mathrm{ch}}=b_{i,t,s}^{\mathrm{dch}}=0$) when the MCS does not visit the FCS (i.e., $b_{i,t,s}^{v}=0$).

(2) $$\begin{array}{cc} \sum_{i\in I}b_{i,t,s}^{v} & \leq 1\;\;\;\;\;\;\;\;\;\;\;\;\;\;\;\;\;\;\;\forall t\in T,\;\forall s\in S \end{array}$$

(3) $$\begin{array}{cc} N_{i,t}^{\mathrm{ch}}+\sum_{s\in S}b_{i,t,s}^{\mathrm{ch}} & \leq N_{i}^{\mathrm{FCS},\mathrm{pole}}\;\;\;\;\;\forall i\in I,\;\forall t\in T \end{array}$$

(4) $$\begin{array}{cc} \sum_{i\in I}b_{i,t,s}^{\mathrm{ch}} & \leq 1-b_{t,s}^{\mathrm{tr}}\;\;\;\;\;\;\;\;\;\forall t\in T,\;\forall s\in S \end{array}$$

(5) $$\begin{array}{cc} \sum_{i\in I}b_{i,t,s}^{\mathrm{dch}} & \leq 1-b_{t,s}^{\mathrm{tr}}\;\;\;\;\;\;\;\;\;\forall t\in T,\;\forall s\in S \end{array}$$

(6) $$\begin{array}{cc} b_{i,t,s}^{\mathrm{ch}}+b_{i,t,s}^{\mathrm{dch}} & \leq b_{i,t,s}^{v}\;\;\;\;\;\;\;\;\;\;\;\;\;\;\forall i\in I,\;\forall t\in T,\;\forall s\in S. \end{array}$$

#### 4.2.2. Routing of MCS

The binary decision variable $b_{ij,t,s}^{c}$ determines the connection status of path i−j of MCS *s* when the MCS departs from node *i* at scheduling time *t* to arrive at node *j*. Equation (7) indicates that MCS *s* has a single path at most in the entire scheduling horizon. Equation (8) expresses the relationship between the visiting status $b_{i,t,s}^{v}$ at FCS *i* and the connection status $b_{ij,t,s}^{c}$ of path i−j for MCS *s*. In Equation (8), the set $I_{i}$ includes nodes adjacent to node *i*. $1_{I^{\mathrm{FCS}}}\left(i\right)$ is the indicator function based on the set $I^{\mathrm{FCS}}$. That is, $1_{I^{\mathrm{FCS}}}\left(i\right)=1$ when $i\in I^{\mathrm{FCS}}$; otherwise, $1_{I^{\mathrm{FCS}}}\left(i\right)=0$. Equation (8) ensures that $b_{ij,t,s}^{c}$ always equals one when the MCS visits and passes through the non-FCS node ($b_{i,t,s}^{v}=1$, $1_{I^{\mathrm{FCS}}}\left(i\right)=0$). However, when the MCS visits the FCS node ($b_{i,t,s}^{v}=1$, $1_{I^{\mathrm{FCS}}}\left(i\right)=1$), $b_{ij,t,s}^{c}$ assumes two values in the following two cases: (i) $b_{ij,t,s}^{c}=1$ when the MCS passes the FCS node and (ii) $b_{ij,t,s}^{c}=0$ when the MCS stays at the FCS node where the MCS is in the charging/discharging mode or idle mode. Equation (9) guarantees that no MCS stays at any node during the traveling time ${\hat{\gamma }}_{ij}$ when the path is connected between nodes *i* and *j*. Here, ${\hat{\gamma }}_{ij}$ is the normalized traveling time with an integer value; it is expressed as ${\hat{\gamma }}_{ij}=\left\lceil \frac{{\hat{\mathrm{tr}}}_{ij}}{\Delta t}\right\rceil$, where ⌈·⌉ is the rounding-up of the ratio of the predicted traveling time ${\hat{\mathrm{tr}}}_{ij}$ to the scheduling time unit Δt. In the connected path i−j, the MCS visits node *j* after ${\hat{\gamma }}_{ij}$ time periods, according to Equation (10). No connected path is allowed while the MCS stays at the FCS node in Equation (11). Equation (12) ensures that the MCS does not travel ($b_{t,s}^{\mathrm{tr}}=0$) when no connected path exists ($b_{ij,t,s}^{c}=0$) and the MCS visits node *i* ($b_{i,t,s}^{v}=1$).

(7) $$\begin{array}{cc} \sum_{i\in I}\sum_{j\in I_{i}}b_{ij,t,s}^{c} & \leq 1\;\;\;\;\;\;\;\;\;\;\;\;\;\;\;\;\;\;\;\;\;\;\;\;\;\;\;\;\;\;\;\forall t\in T,\;\forall s\in S \end{array}$$

(8) $$\begin{array}{cc} b_{i,t,s}^{v}-1_{I^{\mathrm{FCS}}}\left(i\right) & \leq \sum_{j\in I_{i}}b_{ij,t,s}^{c}\leq b_{i,t,s}^{v}\;\;\;\;\;\;\forall i\in I,\;\forall t\in T,\;\forall s\in S \end{array}$$

(9) $$\begin{array}{cc} b_{ij,t,s}^{c}+\frac{1}{\left|I||\Gamma \right|}\sum_{t^{\prime }=t+1}^{t+{\hat{\gamma }}_{ij}-1} & \sum_{j\neq i\in I}b_{j,t^{\prime },s}^{v}\leq 1\;\;\;\;\;\;\;\;\;\;\;\;\;\forall i\in I,\;\forall j\in I_{i},\;\forall t\in T,\;\forall s\in S \end{array}$$

(10) $$\begin{array}{cc} b_{ij,t,s}^{c} & \leq b_{j,t+{\hat{\gamma }}_{ij},s}^{v}\;\;\;\;\;\;\;\;\;\;\;\;\;\;\;\;\;\;\;\;\forall i\in I,\;\forall j\in I_{i},\;\forall t\in T,\;\forall s\in S \end{array}$$

(11) $$\begin{array}{cc} b_{i,t,s}^{v}-\sum_{j\in I_{i}}b_{ij,t,s}^{c} & \leq b_{i,t+1,s}^{v}\;\;\;\;\;\;\;\;\;\;\;\;\;\;\;\;\;\;\;\;\;\;\forall i\in I,\;\forall t\in T,\;\forall s\in S \end{array}$$

(12) $$\begin{array}{cc} b_{t,s}^{\mathrm{tr}}=\sum_{j\in I_{i}}\sum_{i\in I}b_{ij,t,s}^{c}- & \sum_{i\in I}b_{i,t,s}^{v}+1\;\;\;\;\;\;\;\;\;\;\;\;\;\;\;\;\;\;\forall t\in T,\;\forall s\in S. \end{array}$$

#### 4.2.3. Charging and Discharging of MCS

Equation (13) defines the operational dynamics of the state of charge (SOC) for MCS *s* at current scheduling time *t* in terms of the SOC at previous scheduling time t−1, battery capacity ($E_{s}^{\mathrm{MCS},\max}$), charging and discharging efficiency ($\eta_{s}^{\mathrm{ch}}$, $\eta_{s}^{\mathrm{dch}}$), traveling efficiency ($\eta_{s}^{\mathrm{tr}}$), traveling status ($b_{t,s}^{\mathrm{tr}}$), and charging and discharging power ($P_{t,s}^{\mathrm{MCS},\mathrm{ch}}$ and $P_{t,s}^{\mathrm{MCS},\mathrm{dch}}$, respectively). Equation (14) denotes the SOC capacity constraint for the MCS. Equation (15) represents the minimum limit of the SOC with which the MCS can return to the depot after finishing all charging services at scheduling time *T*. Equations (16) and (17) express the limits of the charging ($P_{i,t,s}^{\mathrm{MCS},\mathrm{ch}}$) and discharging ($P_{i,t,s}^{\mathrm{MCS},\mathrm{dch}}$) power of the MCS, respectively. The binary decision variables $b_{i,t,s}^{\mathrm{ch}}$ and $b_{i,t,s}^{\mathrm{dch}}$ determine the charging and discharging statuses of the MCS, respectively. Equation (18) indicates that the charging duration of the MCS is longer than three consecutive scheduling time slots to reduce the frequency of the charging and discharging of the MCS. Equation (19) denotes the sum of the charging and discharging power at all FCSs.

(13) $$\begin{array}{cc} SOC_{t,s}=SOC_{t-1,s} & +\frac{\eta_{s}^{\mathrm{ch}}P_{t,s}^{\mathrm{MCS},\mathrm{ch}}-\eta_{s}^{\mathrm{dch}}P_{t,s}^{\mathrm{MCS},\mathrm{dch}}-\eta_{s}^{\mathrm{tr}}b_{t,s}^{\mathrm{tr}}}{E_{s}^{\mathrm{MCS},\max}}\;\;\forall t\in T,\;\forall s\in S \end{array}$$

(14) $$\begin{array}{cc} SOC_{s}^{\min}\leq & SOC_{t,s}\leq SOC_{s}^{\max}\;\;\;\;\;\;\;\;\;\;\;\;\;\;\;\;\;\;\;\;\;\;\;\;\;\;\;\;\;\;\;\;\;\;\;\;\;\forall t\in T,\;\forall s\in S \end{array}$$

(15) $$\begin{array}{cc} SOC_{T,s}^{r}\leq & SOC_{T,s}\;\;\;\;\;\;\;\;\;\;\;\;\;\;\;\;\;\;\;\;\;\;\;\;\;\;\;\;\;\;\;\;\;\;\;\;\;\;\;\;\;\;\;\;\;\;\;\;\;\;\;\;\;\;\;\;\forall s\in S \end{array}$$

(16) $$\begin{array}{cc} P_{s}^{\mathrm{MCS},\mathrm{ch},\min}b_{i,t,s}^{\mathrm{ch}}\leq & P_{i,t,s}^{\mathrm{MCS},\mathrm{ch}}\leq P_{s}^{\mathrm{MCS},\mathrm{ch},\max}b_{i,t,s}^{\mathrm{ch}}\;\;\;\;\;\;\forall i\in I,\;\forall t\in T,\;\;\forall s\in S \end{array}$$

(17) $$\begin{array}{cc} P_{s}^{\mathrm{MCS},\mathrm{dch},\min}b_{i,t,s}^{\mathrm{dch}}\leq & P_{i,t,s}^{\mathrm{MCS},\mathrm{dch}}\leq P_{s}^{\mathrm{MCS},\mathrm{dch},\max}b_{i,t,s}^{\mathrm{dch}}\;\;\forall i\in I,\;\forall t\in T,\;\forall s\in S \end{array}$$

(18) $$\begin{array}{cc} b_{i,t+1,s}^{\mathrm{ch}} & -b_{i,t,s}^{\mathrm{ch}}\leq \frac{1}{2}\left(b_{i,t+2,s}^{\mathrm{ch}}+b_{i,t+3,s}^{\mathrm{ch}}\right)\;\;\forall i\in I,\;\forall t\in T,\;\forall s\in S \end{array}$$

(19) $$\begin{array}{cc} P_{t,s}^{\mathrm{MCS},\mathrm{ch}}=\sum_{i\in I}P_{i,t,s}^{\mathrm{MCS},\mathrm{ch}}, & \;\;P_{t,s}^{\mathrm{MCS},\mathrm{dch}}=\sum_{i\in I}P_{i,t,s}^{\mathrm{MCS},\mathrm{dch}}\;\;\;\;\;\;\;\;\;\;\;\;\;\;\;\;\;\;\;\;\forall t\in T,\;\forall s\in S. \end{array}$$

### 4.3. Queue Dynamics for EVs at FCS

Equation (20) expresses the number of decreased waiting EVs ($\Delta N_{i,t}$) at FCS *i* and time *t* resulting from the MCS operation until time t−1, which is the difference between the predicted number of waiting EVs (${\hat{N}}_{i,t-1}^{w,\mathrm{Non-MCS}}$) at time t−1 without MCS and the number of waiting EVs ($N_{i,t-1}^{w}$) at t−1. In (20), the value of $N_{i,t-1}^{w}$ is affected by the direct charging of the MCS at time t−1 and the indirect reduction of waiting EVs because of the MCS charging prior to time t−1. Equation (21) represents the number of EVs at FCS *i* and time *t*, which involves MCS charging only until time t−1. The number of charging EVs is determined by the minimum number of EVs and the number of poles in the FCS in (22). Equation (23) represents the number of waiting EVs after charging power from FCS *i* at scheduling time *t*. The number of waiting EVs after charging from both the MCS and FCS at time *t* is given in (24). Equation (25) ensures that the number of EVs charging from the MCS is less than or equal to the number of waiting EVs. The number of EVs charging from the MCS is limited in terms of the discharging status and number of poles for the MCS according to (26).

(20) $$\begin{array}{cc} \Delta N_{i,t} & ={\hat{N}}_{i,t-1}^{w,\mathrm{Non}-\mathrm{MCS}}-N_{i,t-1}^{w}\;\;\;\;\;\;\;\;\;\;\;\;\;\;\;\;\;\;\forall i\in I^{\mathrm{FCS}},\;\forall t\in T \end{array}$$

(21) $$\begin{array}{cc} N_{i,t}^{{}^{\prime }} & =\max\left({\hat{N}}_{i,t}^{\mathrm{Non}-\mathrm{MCS}}-\Delta N_{i,t},0\right)\;\;\;\;\;\;\;\forall i\in I^{\mathrm{FCS}},\;\forall t\in T \end{array}$$

(22) $$\begin{array}{cc} N_{i,t}^{\mathrm{ch}} & =\min\left(N_{i,t}^{{}^{\prime }},N_{i}^{\mathrm{FCS},\mathrm{pole}}\right)\;\;\;\;\;\;\;\;\;\;\;\;\;\;\;\;\;\;\;\forall i\in I^{\mathrm{FCS}},\;\forall t\in T \end{array}$$

(23) $$\begin{array}{cc} N_{i,t}^{w\prime } & =N_{i,t}^{{}^{\prime }}-N_{i,t}^{\mathrm{ch}}\;\;\;\;\;\;\;\;\;\;\;\;\;\;\;\;\;\;\;\;\;\;\;\;\;\;\;\;\;\;\;\;\;\;\;\forall i\in I^{\mathrm{FCS}},\;\forall t\in T \end{array}$$

(24) $$\begin{array}{cc} N_{i,t}^{w} & =N_{i,t}^{w\prime }-\sum_{s\in S}N_{i,t,s}^{\mathrm{MCS}-\mathrm{dch}}\;\;\;\;\;\;\;\;\;\;\;\;\;\;\;\;\;\;\;\forall i\in I^{\mathrm{FCS}},\;\forall t\in T \end{array}$$

(25) $$\begin{array}{cc} \sum_{s\in S}N_{i,t,s}^{\mathrm{MCS}-\mathrm{dch}} & \leq N_{i,t}^{w\prime }\;\;\;\;\;\;\;\;\;\;\;\;\;\;\;\;\;\;\;\;\;\;\;\;\;\;\;\;\;\;\;\;\;\;\;\;\;\;\;\;\;\;\;\;\;\;\forall i\in I^{\mathrm{FCS}},\;\forall t\in T \end{array}$$

(26) $$\begin{array}{cc} b_{i,t,s}^{\mathrm{dch}} & \leq N_{i,t,s}^{\mathrm{MCS}-\mathrm{dch}}\leq N_{s}^{\mathrm{MCS},\mathrm{pole}}b_{i,t,s}^{\mathrm{dch}}\;\;\;\;\;\;\;\forall i\in I^{\mathrm{FCS}},\;\forall t\in T. \end{array}$$

**Figure 3:**
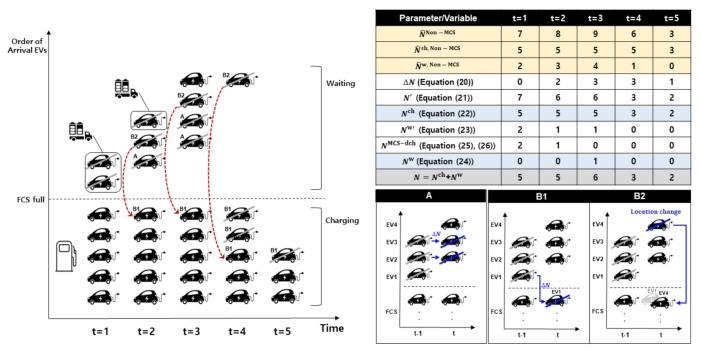
Example illustrating electric vehicle (EV) queue dynamics at fixed charging station (FCS).

Figure 3 illustrates an example of the proposed EV queue dynamics at FCS associated with the MCS operation. We assume that there are no EVs leaving the queue without charging. In this figure, the table provides the values of parameters and variables during the five scheduling periods. These parameters and variables are used in the constraints of EV queue dynamics. The results from the second to fourth rows in this table represent the values of the parameters for the number of EVs, charging EVs, and waiting EVs, respectively. The results from the fifth to tenth rows were obtained using the constraints in Equations (20)–(26). The last row of the table denotes the total number of EVs at the FCS, which is the sum of the charging and waiting EVs. A key part of the EV queue dynamics is the calculation of the number of decreased waiting EVs (ΔN). The image at the bottom right corner in Figure 3 illustrates the impact of ΔN on the number of charging and waiting EVs. This impact is categorized into three cases (Cases A, B1, and B2). In these three cases, we assume that three EVs (EV1, EV2, and EV3) and three EVs (EV2, EV3, and EV4) wait at times t−1 and *t*, respectively. We consider the situation in which three EVs are removed at times t−1 owing to MCS discharging. In Case A, EV2 and EV3 are already removed at time t−1; therefore, EV2 and EV3 in the waiting queue at time *t* are removed as well. Cases B1 and B2 include the scenario where a waiting EV at time t−1 charges power at the FCS at time *t*. In Case B1, given that EV1 was already removed at time t−1, EV1 is also removed from the charging queue at time *t*. In Case B2, EV4 moves from the waiting queue to the charging queue and starts charging at time *t* through the charging pole that is allocated to the removed EV1 in Case B1. As a result, EV2, EV3, and EV4 are removed at time *t* in Cases A, B1, and B2 (ΔN = 3). Finally, the inset on the left side in Figure 3 shows the charging and waiting EV queue dynamics based on the results associated with those listed in the table and the three aforementioned cases.

### 4.4. Power Distribution System Operation

Using linearized DistFlow equations [24], the active and reactive power flows can be written as

(27) $$\begin{array}{cc} P_{hb,t}^{\mathrm{line}} & =\sum_{k\in B_{b}}P_{bk,t}^{\mathrm{line}}+P_{b,t}\;\;\;\;\;\;\forall b,\;h,\;k\in B,\;\forall t\in T \end{array}$$

(28) $$\begin{array}{cc} Q_{hb,t}^{\mathrm{line}} & =\sum_{k\in B_{b}}Q_{bk,t}^{\mathrm{line}}+Q_{b,t}\;\;\;\;\;\forall b,\;h,\;k\in B,\;\forall t\in T. \end{array}$$

On the right-hand side of Equations (27) and (28), the first terms denote the sum of the active power flow ($P_{bk,t}^{\mathrm{line}}$) and reactive power flow ($Q_{bk,t}^{\mathrm{line}}$) from bus *b* to *k* at time *t*, respectively. Here, bus *k* belongs to a set of buses $B_{b}$ that has all buses connected downstream to bus *b*. The second terms represent the active ($P_{b,t}$) and reactive ($Q_{b,t}$) load consumption at bus *b* and time *t*. They are expressed in terms of active and reactive power consumption (${\hat{P}}_{b,t}^{\mathrm{non}-\mathrm{EV}\;\mathrm{load}}$, ${\hat{Q}}_{b,t}^{\mathrm{non}-\mathrm{EV}\;\mathrm{load}}$) for non-EV load, active and reactive power consumption ($P_{b,t}^{\mathrm{EV}\;\mathrm{load}}$, $Q_{b,t}^{\mathrm{EV}\;\mathrm{load}}$) for EV load, and the sum of active charging power ($P_{b,t}^{\mathrm{MCS},\mathrm{ch}}=\sum_{s\in S}P_{b,t,s}^{\mathrm{MCS},\mathrm{ch}}$) and reactive charging or discharging power ($Q_{b,t}^{\mathrm{MCS}}$) for the MCS as expressed in Equations (29) and (30), respectively,

(29) $$\begin{array}{cc} P_{b,t} & ={\hat{P}}_{b,t}^{\mathrm{non}-\mathrm{EV}\;\mathrm{load}}+P_{b,t}^{\mathrm{EV}\;\mathrm{load}}+P_{b,t}^{\mathrm{MCS},\mathrm{ch}}\;\;\;\;\;\;\forall b\in B,\;\forall t\in T \end{array}$$

(30) $$\begin{array}{cc} Q_{b,t} & ={\hat{Q}}_{b,t}^{\mathrm{non}-\mathrm{EV}\;\mathrm{load}}+Q_{b,t}^{\mathrm{EV}\;\mathrm{load}}+Q_{b,t}^{\mathrm{MCS}}\;\;\;\;\;\;\;\;\forall b\in B,\;\forall t\in T. \end{array}$$

Equation (31) denotes the active power consumption for the EV load, which is expressed as the multiplication of the charging rate ($R_{b}^{\mathrm{FCS}}$) per pole of the FCS and the number of charging EVs at bus *b*. The reactive power consumption for the EV load is expressed in terms of its power factor (pf) and active power consumption ($P_{b,t}^{\mathrm{EV}\;\mathrm{load}}$) in Equation (32). The reactive power capability of the MCS is given by Equation (33), which is described in terms of the minimum power factor ($pf_{\min}^{\mathrm{MCS}}$) and the charging/discharging power ($P_{b,t}^{\mathrm{MCS},\mathrm{ch}}$, $P_{b,t}^{\mathrm{MCS},\mathrm{dch}}$) of the MCS. In Equation (34), $P_{b,t,s}^{\mathrm{MCS},\mathrm{dch}}$ supports the load demand of the waiting EVs by MCS *s* and is expressed in terms of the multiplication of the charging rate ($R_{b}^{\mathrm{MCS}}$) and $N_{b,t,s}^{\mathrm{MCS}-\mathrm{dch}}$. Equations (35) and (36) denote the sum of the charging and discharging powers for MCSs at bus *b*, respectively.

(31) $$\begin{array}{cc} P_{b,t}^{\mathrm{EV}\;\mathrm{load}} & =R_{b}^{\mathrm{FCS}}N_{b,t}^{\mathrm{ch}}\;\;\;\;\;\;\;\;\;\;\;\;\;\;\;\;\;\;\;\;\;\;\forall b\in B\cap I^{\mathrm{FCS}},\;\forall t\in T \end{array}$$

(32) $$\begin{array}{cc} Q_{b,t}^{\mathrm{EV}\;\mathrm{load}} & =\sqrt{\frac{1-pf^{2}}{pf^{2}}}P_{b,t}^{\mathrm{EV}\;\mathrm{load}}\;\;\;\;\;\;\forall b\in B\cap I^{\mathrm{FCS}},\;\forall t\in T\\ -\sqrt{\frac{1-{\left(pf_{\min}^{\mathrm{MCS}}\right)}^{2}}{{\left(pf_{\min}^{\mathrm{MCS}}\right)}^{2}}}(P_{b,t}^{\mathrm{MCS},\mathrm{ch}} & +P_{b,t}^{\mathrm{MCS},\mathrm{dch}})\leq Q_{b,t}^{\mathrm{MCS}}\\ \; & \leq \sqrt{\frac{1-{\left(pf_{\min}^{\mathrm{MCS}}\right)}^{2}}{{\left(pf_{\min}^{\mathrm{MCS}}\right)}^{2}}}\left(P_{b,t}^{\mathrm{MCS},\mathrm{ch}}+P_{b,t}^{\mathrm{MCS},\mathrm{dch}}\right) \end{array}$$

(33) $$\begin{array}{cc} \; & \;\;\;\;\;\;\;\;\;\;\;\;\;\;\;\;\;\;\;\;\;\;\;\;\;\;\;\;\;\;\;\;\;\;\;\;\;\;\;\forall b\in B\cap I^{\mathrm{FCS}},\;\forall t\in T \end{array}$$

(34) $$\begin{array}{cc} P_{b,t,s}^{\mathrm{MCS},\mathrm{dch}} & =R_{b}^{\mathrm{MCS}}N_{b,t,s}^{\mathrm{MCS}-\mathrm{dch}}\;\;\;\;\;\;\;\;\forall b\in B\cap I^{\mathrm{FCS}},\;\forall t\in T,\;\forall s\in S \end{array}$$

(35) $$\begin{array}{cc} P_{b,t}^{\mathrm{MCS},\mathrm{ch}} & =\sum_{s\in S}P_{b,t,s}^{\mathrm{MCS},\mathrm{ch}}\;\;\;\;\;\;\;\;\;\;\;\;\;\;\forall b\in B\cap I^{\mathrm{FCS}},\;\forall t\in T \end{array}$$

(36) $$\begin{array}{cc} P_{b,t}^{\mathrm{MCS},\mathrm{dch}} & =\sum_{s\in S}P_{b,t,s}^{\mathrm{MCS},\mathrm{dch}}\;\;\;\;\;\;\;\;\;\;\;\;\forall b\in B\cap I^{\mathrm{FCS}},\;\forall t\in T. \end{array}$$

The voltage drop equation and the limit of the voltage magnitude are, respectively, expressed as follows:

(37) $$\begin{array}{cc} V_{b,t} & =V_{h,t}-\left(\frac{r_{hb}P_{hb,t}^{\mathrm{line}}+x_{hb}Q_{hb,t}^{\mathrm{line}}}{V_{1}}\right)\;\;\;\;\;\;\forall b,h\in B,\;\forall t\in T \end{array}$$

(38) $$\begin{array}{cc} V^{\min} & \leq V_{b,t}\leq V^{\max}\;\;\;\;\;\;\;\;\;\;\;\;\;\;\;\;\;\;\;\;\;\;\;\;\;\;\;\;\;\;\;\;\;\;\;\;\forall b\in B,\;\forall t\in T. \end{array}$$

In Equation (37), $V_{1}$ represents the nominal voltage magnitude (1.0 p.u.) at node 1. In (38), $V^{\min}$ and $V^{\max}$ are set to 0.95 p.u. and 1.05 p.u., respectively.

To formulate the MILP-based optimization problem, the nonlinear equation for the second objective function in Equation (1) and two nonlinear constraints for the EV queue dynamics (Equations (21) and (22)) are, respectively, replaced by the following linearized constraints:

(39) $$\begin{array}{cc} \; & \left\{\begin{array}{c} \Delta V_{b,t}=\left|V_{b,t}-V^{\mathrm{ref}}\right|\\ \Delta V_{b,t}\geq V_{b,t}-V^{\mathrm{ref}}\\ \Delta V_{b,t}\geq V^{\mathrm{ref}}-V_{b,t} \end{array}\right. \end{array}$$

(40) $$\begin{array}{cc} \; & \left\{\begin{array}{c} \Delta N_{i,t}-{\hat{N}}_{i,t}^{\mathrm{Non}-\mathrm{MCS}}\geq -Mb_{i,t}^{a1}\\ {\hat{N}}_{i,t}^{\mathrm{Non}-\mathrm{MCS}}-\Delta N_{i,t}\geq -M\left(1-b_{i,t}^{a1}\right)\\ {\hat{N}}_{i,t}^{\mathrm{Non}-\mathrm{MCS}}-\Delta N_{i,t}\leq N_{i,t}^{{}^{\prime }}\leq {\hat{N}}_{i,t}^{\mathrm{Non}-\mathrm{MCS}}-\Delta N_{i,t}+M\left(1-b_{i,t}^{a1}\right)\\ 0\leq N_{i,t}^{{}^{\prime }}\leq Mb_{i,t}^{a1} \end{array}\right. \end{array}$$

(41) $$\begin{array}{cc} \; & \left\{\begin{array}{c} N_{i}^{\mathrm{FCS},\mathrm{pole}}-N_{i,t}^{{}^{\prime }}\leq Mb_{i,t}^{a2}\\ N_{i,t}^{{}^{\prime }}-N_{i}^{\mathrm{FCS},\mathrm{pole}}\leq M\left(1-b_{i,t}^{a2}\right)\\ N_{i,t}^{{}^{\prime }}-M\left(1-b_{i,t}^{a2}\right)\leq N_{i,t}^{\mathrm{ch}}\leq N_{i,t}^{{}^{\prime }}\\ N_{i}^{\mathrm{FCS},\mathrm{pole}}-Mb_{i,t}^{a2}\leq N_{i,t}^{\mathrm{ch}}\leq N_{i}^{\mathrm{FCS},\mathrm{pole}} \end{array}\right. \end{array}$$

In Equations (40) and (41), $b_{i,t}^{a1}$ and $b_{i,t}^{a2}$ represent auxiliary binary variables and *M* is a large positive constant.

## 5. Numerical Examples

### 5.1. Simulation Setup

**Figure 4:**
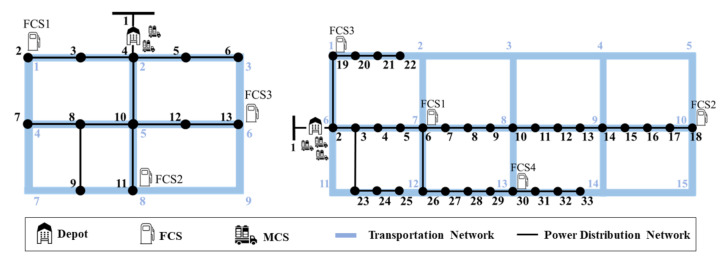
IEEE 13-bus power distribution system with 9-node transportation system and IEEE 33-bus power distribution system with 15-node transportation system.

**Figure 5:**
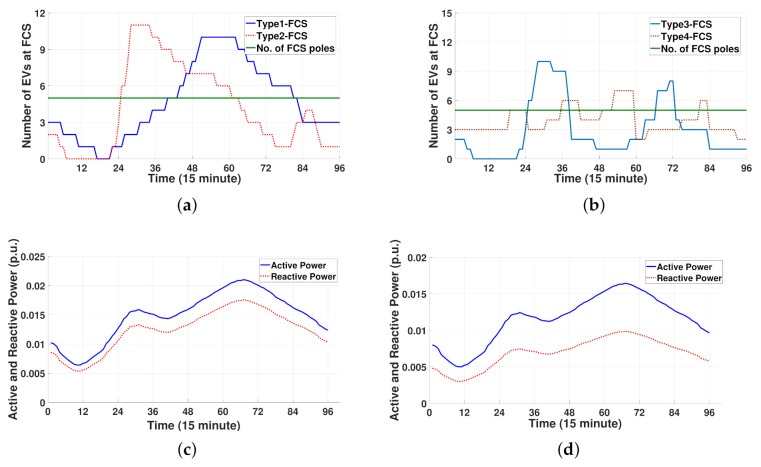
Profiles of operating conditions for transportation and power distribution systems prior to MCS dispatch. (**a**) Total number of EVs at Type1- and Type2-FCSs in 9-node transportation system. (**b**) Total number of EVs at Type3- and Type4-FCSs in 15-node transportation system. (**c**) Active and reactive power consumption in IEEE 13-bus power distribution system. (**d**) Active and reactive power consumption in IEEE 33-bus power distribution system.

In this section, we quantify the performance of the proposed MCS control approach in IEEE 13-bus and 33-bus power distribution systems [25] coupled with 9-node and 15-node transportation systems, respectively, as shown in Figure 4. The simulations were executed for 24 h with a 15 min scheduling resolution (i.e., Δt=15 min). Consequently, the total number of scheduling time slots was T=96. In the two power distribution systems, the base MVA was set to 100 MVA. Each FCS and MCS had five CPs ($N^{\mathrm{FCS},\mathrm{pole}}=5$) and two CPs ($N^{\mathrm{MCS},\mathrm{pole}}=2$), respectively. According to different charging scheduling periods at FCSs, there were four types of FCSs: (i) Type1-FCS with public charger, (ii) Type2-FCS with work charger, (iii) Type3-FCS with public charger, and (iv) Type4-FCS with public charger. In the IEEE 13-bus distribution system with a 9-node transportation system, the depot with two MCSs (MCS1 and MCS2) was located at bus 4. Three FCSs (FCS1∼FCS3) were connected to buses 2, 11, and 13, respectively. FCS1 and FCS2 belong to Type1-FCS, whereas FCS3 belongs to Type2-FCS. In the IEEE 33-bus distribution system with a 15-node transportation system, the depot with three MCSs (MCS1, MCS2, and MCS3) was located at bus 2. Four FCSs (FCS1∼FCS4) were connected to buses 6, 18, 19, and 30, respectively. FCS1, FCS2, FCS3, and FCS4 correspond to Type1-FCS, Type2-FCS, Type3-FCS, and Type4-FCS, respectively. Figure 5a,b shows the predicted number of EVs (${\hat{N}}^{\mathrm{Non}-\mathrm{MCS}}$) at Type1-FCS, Type2-FCS, Type3-FCS, and Type4-FCS prior to MCS dispatch, which was calculated from the results in [26]. In Figure 5a,b, the positive gap between the predicted number of EVs and the number of FCS poles represents the number of waiting EVs. The charging power rate $R_{b}^{\mathrm{FCS}}$ of the FCS at bus *b* was 125 kW and the power factor pf of the EV load at the FCS was 0.95. The total active (${\hat{P}}^{\mathrm{non}-\mathrm{EV}\;\mathrm{load}}$) and reactive power consumption (${\hat{Q}}^{\mathrm{non}-\mathrm{EV}\;\mathrm{load}}$) for non-EV loads in the IEEE 13-bus and 33-bus power distribution systems are illustrated in Figure 5c,d, respectively.

For each MCS *s*, the battery capacity was set to $E_{s}^{\mathrm{MCS},\max}=200$ kWh. The maximum charging/discharging power and the minimum charging/discharging power were identically set to $P_{s}^{\mathrm{MCS},\mathrm{ch},\max}=P_{s}^{\mathrm{MCS},\mathrm{dch},\max}=125$ kW and $P_{s}^{\mathrm{MCS},\mathrm{ch},\min}=P_{s}^{\mathrm{MCS},\mathrm{dch},\min}=40$ kW. The initial, minimum, and maximum SOCs were selected as 0.6, 0.2, and 0.8, respectively, and the charging and discharging efficiencies $\eta_{s}^{\mathrm{ch}}$ and $\eta_{s}^{\mathrm{dch}}$ were both 0.95. The minimum SOC for returning to the depot at time *T* was set to $SOC_{s,T}^{r}=0.6$. The traveling efficiency was set to $\eta_{s}^{\mathrm{tr}}=2$ kW/Δt. The charging power rate $R_{b}^{\mathrm{MCS}}$ of the MCS at bus *b* was 125 kW and the minimum power factor $pf_{\min}^{\mathrm{MCS}}$ of the MCS was 0.95. We assumed that MCSs supply power only to waiting EVs that cannot be charged from the FCS owing to the limited number of FCS poles. The normalized traveling time between nodes *i* and *j* was set to ${\hat{\gamma }}_{ij}=1$. The proposed MCS control algorithm was simulated in a computer (IntelCore i7-4790 CPU clocking at 3.6 GHz and 4 GB of RAM) using IBM ILOG CPLEX Optimization Studio 12.8 solver through MATLAB R2020a.

In sum, our simulation studies were conducted in the following two simulation models: (i) IEEE 13-bus with 9-node transportation system and (ii) IEEE 33-bus with 15-node transportation system. IEEE 13-bus and 33-bus systems represent the simulation models of power distribution systems, whereas 9-node and 15-node systems represent the simulation models of transportation systems. The proposed optimization algorithm in these simulation models was implemented by MATLAB. All parameters and data for the simulation models of power distribution and transportation systems including the MCS operation were used in the proposed optimization algorithm that is illustrated in Section 4. Using the CPLEX optimization solver, the proposed optimization algorithm with the aforementioned parameters and data was simulated to calculate the optimal day-ahead schedules of the routing and charging/discharging of MCSs in the transportation and power distribution systems, respectively.

### 5.2. Simulation Results for the IEEE 13-Bus Power Distribution System with 9-Node Transportation System

**Figure 6:**
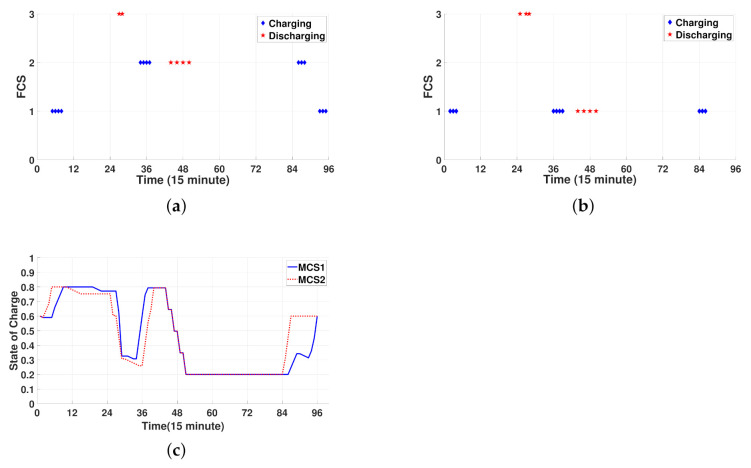
Spatial-temporal schedule for charging and discharging of MCSs. (**a**) Location of MCS1. (**b**) Location of MCS2. (**c**) State of charge (SOC) for MCS1 and MCS2.

Figure 6a–c shows the operating schedule of two MCSs for 24 h. Note from Figure 6a that MCS1 first visits FCS1 and charges power during the period [1:00 a.m., 2:00 a.m.]. After charging power, MCS1 moves from FCS1 to FCS3 and discharges power to the waiting EVs at FCS3 during the period [6:30 a.m., 7:00 a.m.]. In this figure, the fact that there are no charging and discharging marks implies that the MCSs stay at the FCSs without charging and discharging or move to other FCSs. Then, MCS1 moves from FCS3 to FCS2 and performs two tasks: (i) charging power through FCS2 during the period [8:15 a.m., 9:15 a.m.] and (ii) discharging power to the waiting EVs at FCS2 at four time slots ([10:45 a.m., 11:00 a.m.], [11:15 a.m., 11:30 a.m.], [11:45 a.m., 12:00 p.m.], and [12:15 p.m., 12:30 a.m.]). Thereafter, MCS1 stays at FCS2 without charging and discharging until 9:15 p.m. and charges power during the period [9:15 p.m., 10:00 p.m.]. Finally, MCS1 moves from FCS2 to FCS1 and recharges power through FCS1 to obtain the minimum SOC level ($SOC_{s,T}^{r}=0.6$). As shown in Figure 6b, MCS2 conducts the movement, charging, and discharging processes, similar to MCS1. In contrast to the charging and discharging processes of MCS1 at FCS2, MCS2 is dispatched to FCS1, where MCS2 charges power through FCS1 during the period [8:45 a.m., 9:45 a.m.] and discharges power to the waiting EVs at FCS1 during the same four time slots as MCS1. Figure 6c shows the SOC schedule for MCS1 and MCS2 for 24 h. Through a comparison of the results shown in Figure 6a–c, we verify that the SOCs of the MCSs increase (or decrease) as the MCSs charge (or discharge) power. In addition, Figure 6c shows that the SOC decreases less sharply in some scheduling periods than in the other periods. These scheduling periods correspond to the periods when the MCSs move from one FCS to another. For example, the SOC of MCS2 gradually decreases during the period [7:00 a.m., 8:45 a.m.] because MCS2 moves from FCS3 to FCS1 during this period.

Table 1 shows the results of the routing and charging/discharging for MCS1 during the scheduling period [6:45 a.m., 8:30 a.m.] (the time slot [28, 34]). In this period, MCS1 visits nodes 6, 9, and 8 sequentially so that the binary visiting status variable ($b_{i,t,s}^{v}$) becomes one. MCS1 discharges power to the waiting EVs at FCS3 connected to node 6 during the period [6:45 a.m., 7:00 a.m.] (i.e., $b_{6,28,1}^{\mathrm{dch}}=1$), and the SOC of MCS1 becomes 0.32. Then, MCS1 remains in the idle mode during the period [7:00 a.m., 7:30 a.m.] at the same node. During the period [7:30 a.m., 8:00 a.m.], MCS1 moves from node 6 to node 8 having FCS2 via node 9, and the SOC of MCS1 decreases from 0.32 to 0.30 owing to its power consumption for the traveling. In these two consecutive traveling periods with normalized traveling times (${\hat{\gamma }}_{69}=1$ and ${\hat{\gamma }}_{98}=1$), the binary connection status becomes one (i.e., $b_{69,31,1}^{c}=1$ and $b_{98,32,1}^{c}=1$). When MCS1 visits FCS2 connected to node 8, it first reaches the idle mode at 8:00 a.m. and starts charging power at 8:15 a.m. to supply power to the EVs that wait at FCS2.

**Figure 7:**
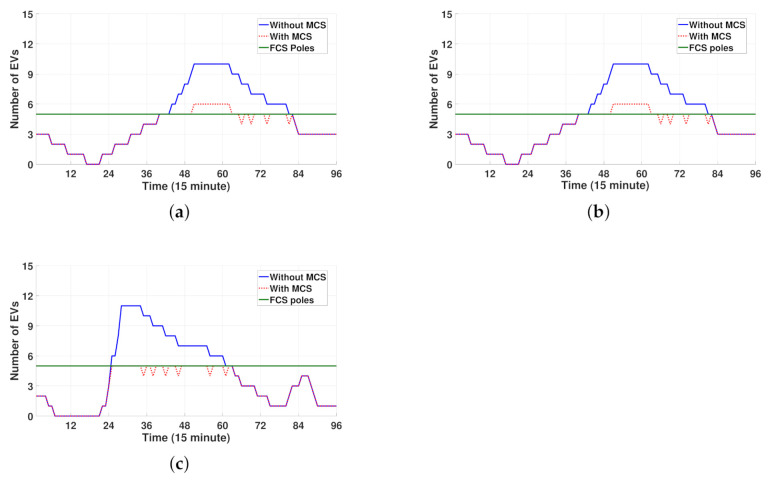
Comparison of the number of EVs without and with MCS between three FCSs. (**a**) FCS1. (**b**) FCS2. (**c**) FCS3.

Figure 7a–c shows the total number of EVs at three FCSs without and with MCS during 24 h. In each figure, a number of EVs greater than the number of poles at the FCS indicates the number of waiting EVs at the FCS. Note from Figure 7a–c that the number of waiting EVs at the three FCSs decreases significantly after MCS dispatch. Another observation is that the distributions of waiting EVs at FCS1 and FCS2 are identical. This is because MCS2 and MCS1 discharge the same amount of power to the waiting EVs at FCS1 and FCS2, respectively, in the same scheduling periods, as shown in Figure 6a,b, respectively. However, compared to the results shown in Figure 6a,b, no waiting EVs are identified at FCS3 after the MCS dispatch, as shown in Figure 6c. This is because both MCS1 and MCS2 are dispatched to FCS3 simultaneously, thereby leading to a greater reduction in waiting EVs at FCS3 than the reduction of waiting EVs at FCS1 and FCS2 owing to individual MCS dispatch.

**Figure 8:**
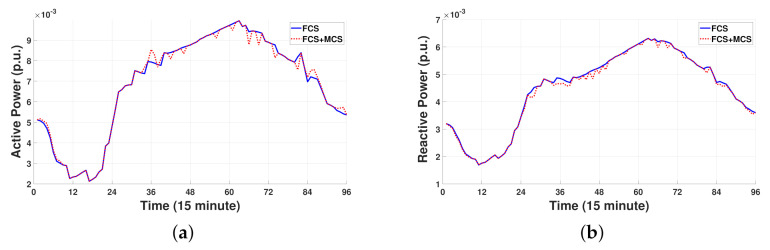
Comparison of total active and reactive power consumption at three FCSs with and without MCSs. (**a**) Active power. (**b**) Reactive power.

Figure 8a,b shows the total sum of active and reactive power consumption for three FCSs without and with MCSs during 24 h. Note from Figure 8a that, in general, the total active power consumption at FCSs with MCSs is less than or equal to the total active power consumption at FCSs without MCSs. This reduction in active power consumption at the FCSs is associated with a reduction in waiting EVs owing to the MCS power supply. However, as shown in Figure 8a, the total active power consumption at FCSs with MCSs is larger than the total active power consumption at FCSs without MCSs in the following three scheduling periods: 9:00 a.m., 9:00 p.m., and 12:00 a.m. These scheduling periods are consistent with the charging periods of the MCSs when the MCSs charge power from the grid through the remaining port of the FCS for discharging power to the waiting EVs or keeping the SOC level with which the MCSs can return to the depot. By contrast, note from Figure 8b that the total reactive power consumption at FCSs with MCSs is less than or equal to the total reactive power consumption at FCSs without MCSs. This observation is derived from the fact that the amount of reactive power injection of the MCSs into the grid becomes larger than the amount of reactive power absorption of the MCSs from the grid to achieve the normal voltage level.

**Figure 9:**
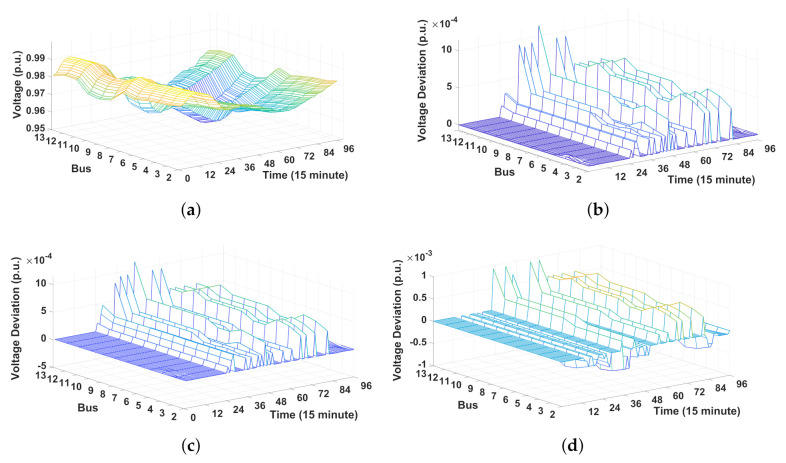
Voltage profile and voltage deviation for 12 buses during 24 h. (**a**) Voltage magnitude after the MCS dispatch. (**b**) Voltage deviation ($\omega_{1}=0.2$, $\omega_{2}=0.8$). (**c**) Voltage deviation ($\omega_{1}=0.5$, $\omega_{2}=0.5$). (**d**) Voltage deviation ($\omega_{1}=1$, $\omega_{2}=0$).

Figure 9a–d shows the voltage profiles and voltage deviation for 32 buses during a 24 h period. Given that the voltage magnitude at bus 1 is fixed at 1 p.u., it is omitted in these figures. Here, the voltage deviation represents the gap between the voltage magnitudes with and without MCS. Therefore, a positive voltage deviation implies that the MCS can mitigate lower voltage limit violations by increasing the voltage magnitude through the injection of reactive power into the grid. Figure 9a shows the voltage magnitude results including the MCSs where the voltage magnitudes are maintained within the lower half of the allowable voltage range [0.95 p.u., 1.05 p.u.]. Figure 9b–d shows voltage deviations due to the MCS operation in terms of weights in the multi-objective function (1). By comparing the results between these three figures, we observe that the voltage deviation increases as the weight $\omega_{2}$ for the voltage deviation from voltage magnitude reference increases. This observation implies that a higher $\omega_{2}$ allows the voltage magnitude to become higher and closer to 1 p.u., thereby preventing the violation of voltage at the lowest level with 0.95 p.u.

**Figure 10:**
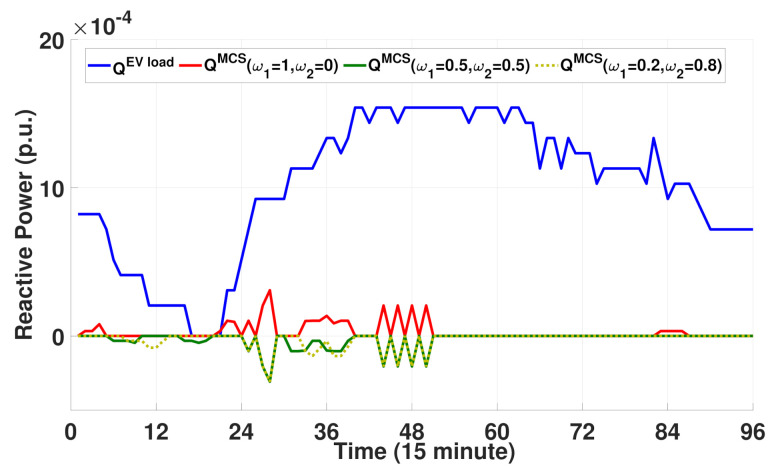
Reactive power consumption schedule for EVs and MCSs with different weights.

Figure 10 compares the reactive power consumption schedule for EVs and MCSs in terms of the weights. Note from this figure that the plot for the EV reactive power load is positive because EVs only consume reactive power from the grid without injecting reactive power into the grid. By contrast, the MCSs can absorb or inject reactive power from/to the grid to maintain a normal voltage profile along the distribution feeder. With a pair of weights $\left\{\omega_{1}=1,\;\omega_{2}=0\right\}$, the proposed MCS optimization algorithm focuses only on reducing the number of waiting EVs without considering the decrease in voltage deviation. Therefore, similar to EVs, the reactive power is always consumed by the MCSs with $\omega_{1}=1$ and $\omega_{2}=0$. However, note from Figure 10 that, as the weight $\omega_{2}$ increases ($\left\{\omega_{1}=0.5,\;\omega_{2}=0.5\right\}$ and $\left\{\omega_{1}=0.2,\;\omega_{2}=0.8\right\}$), the MCSs inject more reactive power into the grid (i.e., negative reactive power consumption) to maintain the desired voltage level. In addition to the results shown in Figure 9b–d, the results in Figure 10 demonstrate that the proposed approach can maintain power distribution system operations with good voltage quality by exploiting the reactive power capability of the MCSs.

**Figure 11:**
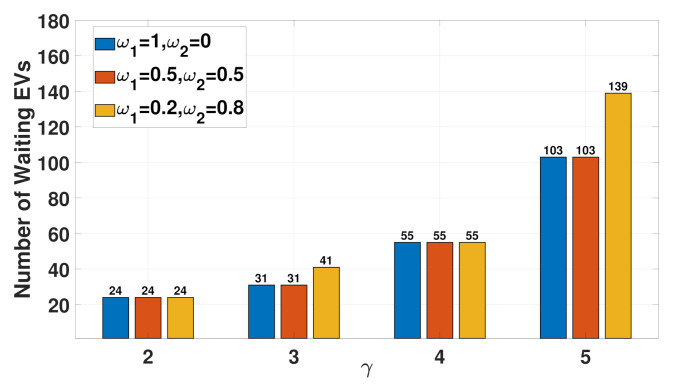
Comparison of the total number of waiting EVs at three FCSs in terms of different weights and normalized traveling times.

Figure 11 shows the results obtained for the number of waiting EVs at three FCSs under four different traffic conditions each of which presents three different weights. The traffic conditions are defined as the normalized traveling time ${\hat{\gamma }}_{ij}$ between nodes *i* and *j*, and a higher ${\hat{\gamma }}_{ij}$ implies more traffic congestion. In Figure 11, four different traffic conditions correspond to ${\hat{\gamma }}_{ij}=2$, ${\hat{\gamma }}_{ij}=3$, ${\hat{\gamma }}_{ij}=4$, and ${\hat{\gamma }}_{ij}=5$ for all i,j∈I. As expected, this figure shows that as ${\hat{\gamma }}_{ij}$ increases, the number of waiting EVs also increases. This is natural because the MCSs are dispatched to their desired FCSs at a slower rate with higher traffic congestion, thereby leading to an increase in the number of waiting EVs. Another observation is that, in general, the greater the weight $\omega_{2}$ (or the lower the weight $\omega_{1}$), the larger the number of waiting EVs. This observation justifies the trade-off relationship between the number of waiting EVs and the voltage deviation in the multi-objective function (1). In other words, a greater $\omega_{2}$ yields a flatter voltage profile with a more positive voltage deviation (as shown in Figure 9b–d) at the expense of an increase in the number of waiting EVs (as shown in Figure 11).

### 5.3. Simulation Results for the IEEE 33-Bus Power Distribution System with 15-Node Transportation System

**Figure 12:**
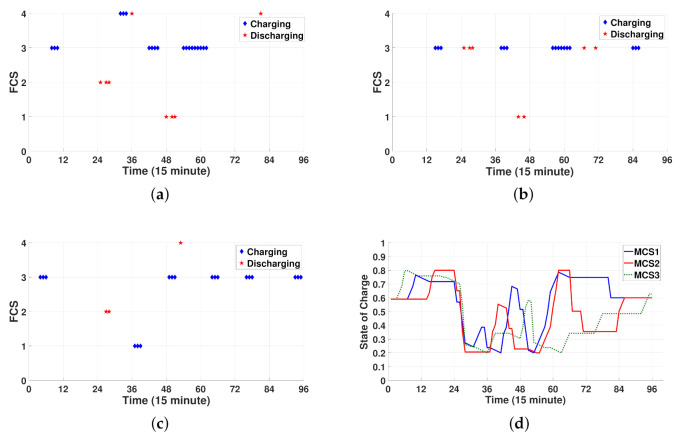
Spatial-temporal schedule for charging and discharging of MCSs. (**a**) Location of MCS1. (**b**) Location of MCS2. (**c**) Location of MCS3. (**d**) SOC for MCS1, MCS2, and MCS3.

Figure 12a–d shows the operating schedule of three MCSs for 24 h. Note from Figure 12a–c that three MCSs visit FCSs for charging and discharging via the following routes: (i) FCS3→FCS2→FCS4→FCS3→FCS1→FCS3→FCS4 for MCS1, (ii) FCS3→FCS1→ FCS3 for MCS2, and (iii) FCS3→FCS2→FCS1→FCS3→FCS4→FCS3 for MCS3. Figure 12d shows the SOC schedule for three MCSs for 24 h, which is consistent with the results in Figure 12a–c. That is, the charging and discharging processes correspond to the increase and decrease in the SOC, respectively. In addition, the SOC curve for traveling is verified to decrease at a slower rate than the SOC curve for discharging. Furthermore, note from Figure 12d that three MCSs can maintain the desired SOC level ($SOC_{T,s}\geq SOC_{s}^{r}=0.6$) at the last scheduling period T=96 to return to the depot successfully.

**Figure 13:**
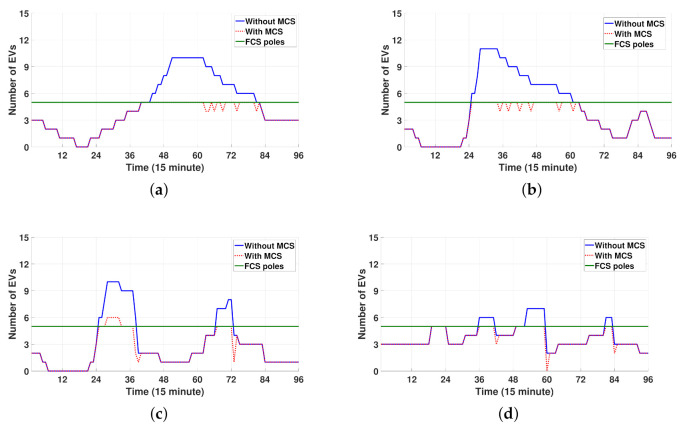
Comparison of the number of EVs without and with MCS between four FCSs. (**a**) FCS1. (**b**) FCS2. (**c**) FCS3. (**d**) FCS4.

Figure 13a–d compares the total number of EVs at four FCSs without and with MCS during 24 h. Note from these figures that the number of waiting EVs at four FCSs decreases significantly owing to the MCS discharging to waiting EVs at discharging scheduling periods as shown in Figure 12a–c. Another observation is that FCS3 still has waiting EVs during the scheduling period [6:45 a.m., 8:00 a.m.]. This is because only a single MCS (MCS2) is dispatched to FCS3 during this period, as illustrated in Figure 12a–c.

**Figure 14:**
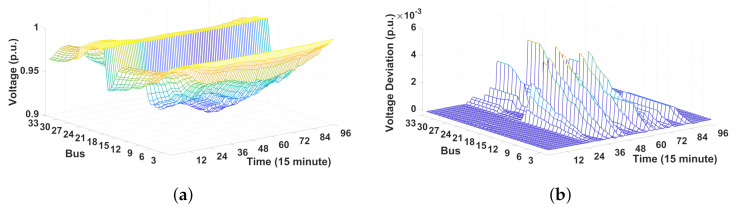
Voltage profile and voltage deviation for 32 buses during 24 h. (**a**) Voltage magnitude after the MCS dispatch. (**b**) Voltage deviation ($\omega_{1}=0.5$, $\omega_{2}=0.5$).

Figure 14a,b shows voltage profiles and voltage deviations for 32 buses during 24 h. In these figures, the minimum and maximum limits of the voltage magnitude are set to $V^{\min}=0.9$ p.u. and $V^{\max}=1.05$ p.u., respectively. Similar to the voltage results for the IEEE 13-bus system depicted in Figure 9a, Figure 14a shows that the voltage magnitudes after the MCS dispatch are maintained within the allowable voltage range. Furthermore, as shown in Figure 9b, the voltage magnitudes at most of the buses after MCS dispatch become larger. This phenomenon is consistent with the results in the IEEE 13-bus system, which prevents an undervoltage at the minimum voltage limit.

**Figure 15:**
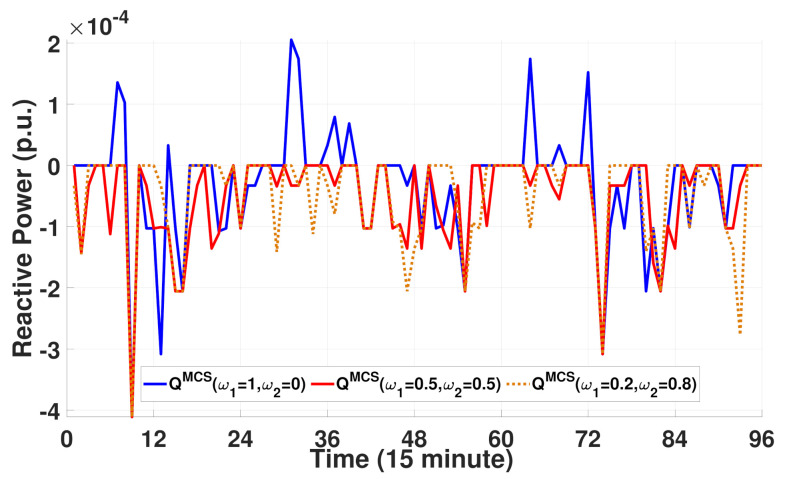
Total reactive power consumption scheduled for three MCSs with different weights.

Figure 15 compares the total reactive power consumption scheduled for three MCSs under varying weights. As expected, a higher weight $\omega_{2}$ (or a lower weight $\omega_{1}$) leads to a more reactive power injection into the grid, which is consistent with the results for the IEEE 13-bus system. Consequently, the reactive power capability of the MCS increases the voltage profile along the distribution feeder. The reactive power injection of the MCS under $\omega_{1}=0.5$ and $\omega_{2}=0.5$ in Figure 15 yields a positive voltage deviation, as shown in Figure 14b, and maintains good voltage quality.

**Figure 16:**
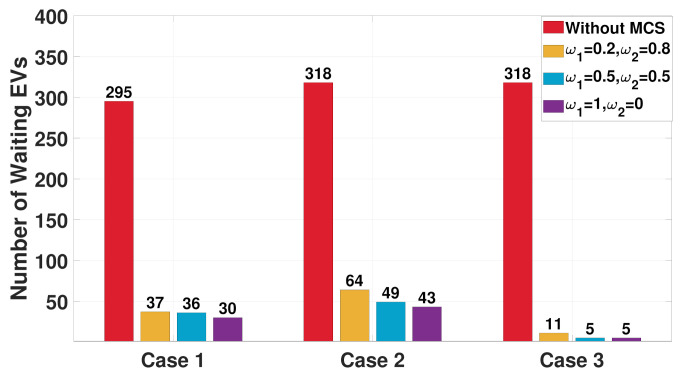
Comparison of the total number of waiting EVs with and without MCS in terms of different weights for three cases.

Figure 16 compares the number of waiting EVs without and with MCS and three different weights in the following three cases:

- Case 1: three FCSs (FCS1∼3) and two MCSs
- Case 2: four FCSs and two MCSs
- Case 3: four FCSs and three MCSs

Note first from this figure that the number of waiting EVs can decrease significantly for the three cases after the MCS dispatch. Note also that a larger weight $\omega_{1}$ results in a smaller number of waiting EVs owing to the greater importance given to the reduction of waiting EVs in the objective function. By comparing Cases 1 and 2, we verify that as the number of FCSs increases with the same number of MCSs, the number of waiting EVs increases as well. The impact of the number of MCSs on the reduction of waiting EVs was investigated by comparing Cases 2 and 3. Note from Figure 16 that, under the situation with the same number of FCSs, an increasing number of MCSs can lead to a significant reduction in the number of waiting EVs.

## 6. Conclusions

In this study, we propose a day-ahead optimal scheduling algorithm for truck-mounted MCSs to reduce the number of waiting EVs at the FCS through the dispatch of the MCSs while maintaining the allowable voltage level in power distribution networks. In mutually coupled transportation and power distribution systems, the proposed optimization algorithm conducts the following two tasks simultaneously: (i) the road routing scheduling of the MCS in the transportation network and (ii) the charging and discharging scheduling of the MCS in the power distribution network that involves realistic operating conditions such as active/reactive power flow and consumption, reactive power absorption/injection from/to the grid, and voltage quality. Routing scheduling considers the statuses of visit, traveling, and traveling path connection of the MCS in the transportation network, along with the interdependent status of charging/discharging and visit of the MCS in the coupled transportation and power distribution networks. The charging and discharging scheduling involves the SOC dynamics for the MCS and the charging and discharging status of the MCS, both of which are coupled with the queue dynamics at the FCS, including charging and waiting EVs. Numerical examples under different penalty weights and traffic conditions confirm that the proposed algorithm can successfully reduce the number of waiting EVs while maintaining an acceptable voltage profile, owing to the reactive power capability of the MCS.

In future work, we plan to evaluate the performance of the proposed MCS optimization algorithm in large-scale transportation and power distribution networks equipped with various distributed energy resources, such as solar photovoltaic systems and ESSs. In addition, the proposed algorithm will be extended to a universal framework where the MCS and voltage regulators, such as an on-load tap changer and a capacitor bank, can cooperate to maintain the voltage quality along the power distribution feeders.

## Figures and Tables

**Table 1:** Schedule for the routing and charging/discharging of MCS1 during the scheduling period [6:45 a.m., 8:30 a.m.].

Time	Node (i,j)	State	SOC	$b_{\mathrm{ij},t,s}^{c}$	$b_{i,t,s}^{v}$	$b_{i,t,s}^{\mathrm{ch}}$	$b_{i,t,s}^{\mathrm{dch}}$	${\hat{\gamma }}_{\mathrm{ij}}$
28	6	Discharging	0.32	-	$b_{6,28,1}^{v}=1$	-	$b_{6,28,1}^{\mathrm{dch}}=1$	-
29	6	Idle	0.32	-	$b_{6,29,1}^{v}=1$	-	-	-
30	6	Idle	0.32	-	$b_{6,30,1}^{v}=1$	-	-	-
31	6 → 9	Travel	0.31	$b_{69,31,1}^{c}=1$	$b_{6,31,1}^{v}=1$	-	-	${\hat{\gamma }}_{69}=1$
32	9 → 8	Travel	0.30	$b_{98,32,1}^{c}=1$	$b_{9,32,1}^{v}=1$	-	-	${\hat{\gamma }}_{98}=1$
33	8	Idle	0.30	-	$b_{8,33,1}^{v}=1$	-	-	-
34	8	Charging	0.45	-	$b_{8,34,1}^{v}=1$	$b_{8,34,1}^{\mathrm{ch}}=1$	-	-

## Data Availability

Not applicable.

## Abbreviations

The following abbreviations are used in this manuscript:

EV	Electric Vehicle
FCS	Fixed Charging Station
MCS	Mobile Charging Station
CPTS	Cyber-Physical Transportation System
ESS	Energy Storage System
TESS	Truck-Mounted ESS
TG	Truck-Mounted Generator
SOC	State of Charge
MILP	Mixed-Integer Linear Programming

## Nomenclature

The main notations are summarized below. Other undefined symbols are explained in the text.

*Sets*	
I	Set of nodes in the transportation network
$I^{\mathrm{FCS}}$	Set of nodes with FCS
$I^{\mathrm{Non}-\mathrm{FCS}}$	Set of nodes without FCS
B	Set of buses in the distribution power network
$B^{\mathrm{FCS}}$	Set of buses with FCS
$B^{\mathrm{Non}-\mathrm{FCS}}$	Set of buses without FCS
S	Set of MCSs
T	Set of scheduling horizon with 15 min resolution
*Subscripts*	
*i*	Subscript for nodes in the transportation network
*b*	Subscript for buses in the distribution power network
*s*	Subscript for MCS
*t*	Subscript for time in the set of scheduling horizons T with 15 min resolution
ij	Subscript for path from node *i* to node *j* in the transportation network
hb	Subscript for line from bus *h* to bus *b* in the distribution power network
*Parameters*	
Δt	15-minute time slot
$N_{i}^{\mathrm{FCS},\mathrm{pole}}$	Number of poles for FCS *i*
$N_{s}^{\mathrm{MCS},\mathrm{pole}}$	Number of poles for MCS *s*
${\hat{N}}_{i,t}^{\mathrm{Non}-\mathrm{MCS}}$	Predicted number of EVs at FCS *i* at time *t* when no MCS is dispatched
${\hat{N}}_{i,t}^{w,\mathrm{Non}-\mathrm{MCS}}$	Predicted number of waiting EVs at FCS *i* at time *t* when no MCS is dispatched
${\hat{\mathrm{tr}}}_{ij}$	Predicted traveling time from node *i* to node *j*
${\hat{\gamma }}_{ij}$	Predicted traveling time unit from node *i* to node *j* that is normalized by Δt
$\eta_{s}^{\mathrm{ch}}$	Charging efficiency of MCS *s*
$\eta_{s}^{\mathrm{dch}}$	Discharging efficiency of MCS *s*
$\eta_{s}^{\mathrm{tr}}$	Traveling power consumption efficiency of MCS *s* per Δt
$E_{s}^{\mathrm{MCS},\max}$	Battery capacity of MCS *s*
$P_{s}^{\mathrm{MCS},\mathrm{ch},\max(\min)}$	Maximum(minimum) charging power of MCS *s*
$P_{s}^{\mathrm{MCS},\mathrm{dch},\max(\min)}$	Maximum(minimum) discharging power of MCS *s*
$SOC_{s}^{\max(\min)}$	Maximum(minimum) state of charge of MCS *s*
$SOC_{T,s}^{r}$	Minimum state of charge of MCS *s* at time *T*
$R_{b}^{\mathrm{FCS}}$	Charging rate of FCS at bus *b*
$R_{b}^{\mathrm{MCS}}$	Charging rate of MCS at bus *b*
$\hat{P}{\left(\hat{Q}\right)}_{b,t}^{\mathrm{non}-\mathrm{EV}\;\mathrm{load}}$	Predicted active(reactive) power consumption of non-EV load at bus *t*
	and time *t*
pf	Power factor of EV load
$pf_{\min}^{\mathrm{MCS}}$	Minimum power factor of MCS
$r_{hb}$	Resistance of line from bus *h* to bus *b*
$x_{hb}$	Reactance of line from bus *h* to bus *b*
$V^{\max(\min)}$	Maximum(minimum) voltage magnitude
*Binary decision variables*	
$b_{i,t,s}^{v}$	Binary visiting state of MCS *s* at node *i* at time *t*: “1” for visit, “0” for non-visit
$b_{i,t,s}^{\mathrm{ch}}$	Binary charging state of MCS *s* at node *i* at time *t*: “1” for charging,
	“0” for non-charging
$b_{i,t,s}^{\mathrm{dch}}$	Binary discharging state of MCS *s* at node *i* at time *t*: “1” for discharging,
	“0” for non-discharging
$b_{t,s}^{\mathrm{tr}}$	Binary traveling state of MCS *s* at time *t*: “1” for traveling, “0” for non-traveling
$b_{ij,t,s}^{c}$	Binary path connection state of MCS *s* from node *i* to node *j* at time *t*:
	“1” for connection, “0” for non-connection
*Integer decision variables*	
$N_{i,t}^{\mathrm{ch}}$	Number of charging EVs at FCS *i* at time *t*
$N_{i,t}^{w}$	Number of waiting EVs at FCS *i* at time *t*
$N_{i,t}$	Number of EVs at FCS *i* at time *t* ($N_{i,t}=N_{i,t}^{\mathrm{ch}}+N_{i,t}^{w}$)
$N_{i,t,s}^{\mathrm{MCS}-\mathrm{dch}}$	Number of EVs that MCS *s* discharges at FCS *i* at time *t*
*Continuous decision variables*	
$P_{b,t,s}^{\mathrm{MCS},\mathrm{ch}}$	Active charging power of MCS *s* at bus *b* and time *t*
$P_{b,t,s}^{\mathrm{MCS},\mathrm{dch}}$	Active discharging power of MCS *s* at bus *b* and time *t*
$P{\left(Q\right)}_{b,t}^{\mathrm{EV}\;\mathrm{load}}$	Active(reactive) charging power of EVs from FCS at bus *b* at time *t*
$Q_{b,t}^{\mathrm{MCS}}$	Injected/absorbed reactive power from MCS at bus *b* at time *t*
$SOC_{t,s}$	State of charge of MCS *s* at time *t*
$P{\left(Q\right)}_{hb,t}^{\mathrm{line}}$	Active(reactive) power flow from bus *h* to bus *b* at time *t*
$P{\left(Q\right)}_{b,t}$	Nodal active(reactive) power at bus *b* at time *t*
$V_{b,t}$	Voltage magnitude at bus *b* at time *t*
